# Supplementary material for: Nature of 2D XY antiferromagnetism in a van der Waals monolayer
Source: Nat Commun. 2025 Dec 1;17:60. doi: 10.1038/s41467-025-66672-1 (PMC12770581; doi:10.1038/s41467-025-66672-1)
Supplement: Supplementary file 1 — Supplementary Information [file 41467_2025_66672_MOESM1_ESM.pdf]

Supplementary information

Nature of 2D XY antiferromagnetism in a van der Waals monolayer

Cheol-Yeon Cheon[1, 2], Volodymyr Multian[1, 2], Kenji Watanabe[3], Takashi Taniguchi[4], Alberto F. Morpurgo[1, 2 *], and Dmitry Lebedev[1, 2 *]

1. Department of Quantum Matter Physics, University of Geneva, 24 Quai Ernest Ansermet, CH-1211 Geneva, Switzerland
2. Department of Applied Physics, University of Geneva, 24 Quai Ernest Ansermet, Geneva, CH-1211 Switzerland
3. Research Center for Electronic and Optical Materials, National Institute for Materials Science, 1-1 Namiki, Tsukuba 305-0044, Japan
4. Research Center for Materials Nanoarchitectonics, National Institute for Materials Science, 1-1 Namiki, Tsukuba 305-0044, Japan

**Correspondence should be addressed to: alberto.morpurgo@unige.ch and dmitry.lebedev@unige.ch*

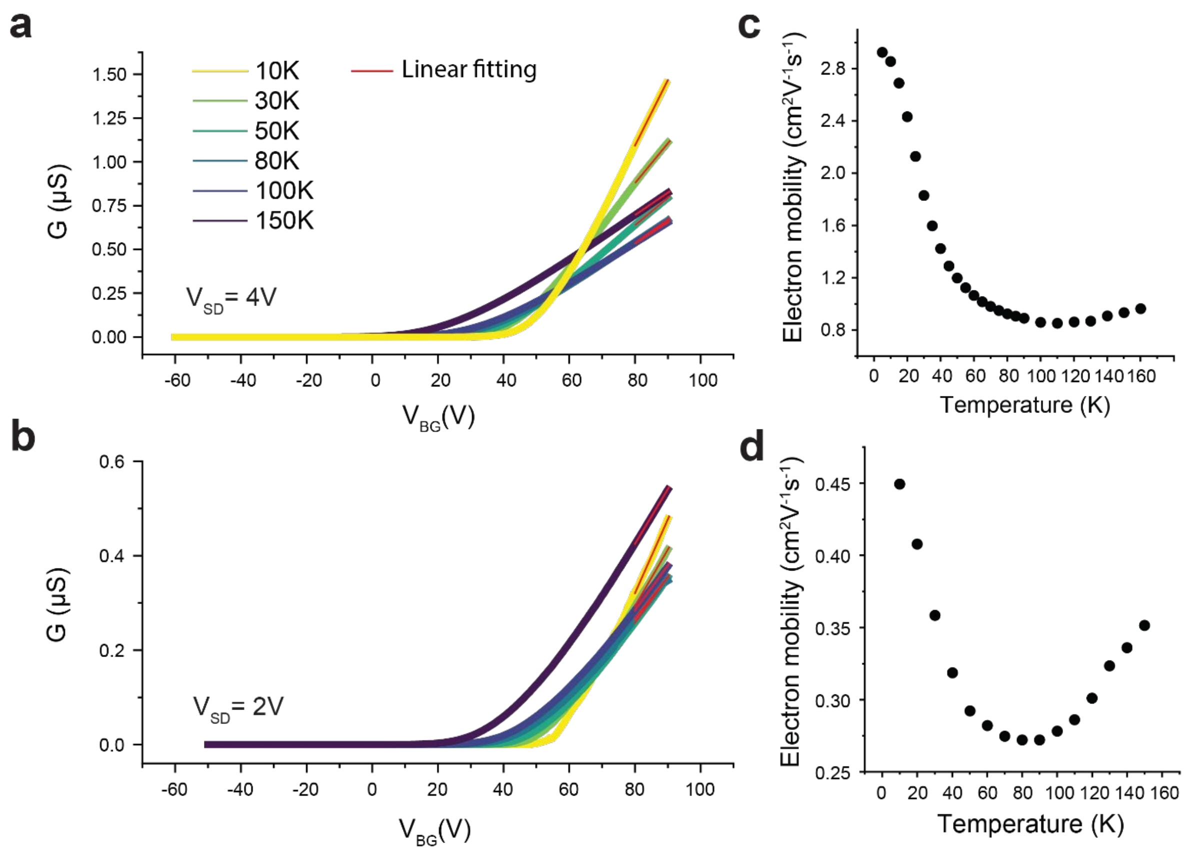

Figure S1. **Estimation of low-temperature field-effect electron mobility of 6L and 1L $NiPS_3$.** **a-b**. Transfer curves of 6L (**a**) and 1L (**b**) measured at the temperature range from 10 K to 150 K. The red lines are the linear fitting at high back-gate voltage (V_{BG}). **c-d**. 2-terminal field effect electron mobility for 6L (**c**) and 1L (**d**) as a function of temperature. Mobility was calculated based on its transconductance (dG/dV_{BG}) in linear regime (V_{BG}=80-90 V for both 6L and 1L), using $\frac{L}{W*C_{\text{total}}}\left(\frac{dG}{dV_{\text{BG}}}\right)$ where L, W, and C_{total} corresponds to the length and width of the conducting channel and the total capacitance of SiO_2 (ε_r= 3.9, thickness: 285 nm) and h-BN (ε_r= 3.76, thickness: 30 nm) respectively.

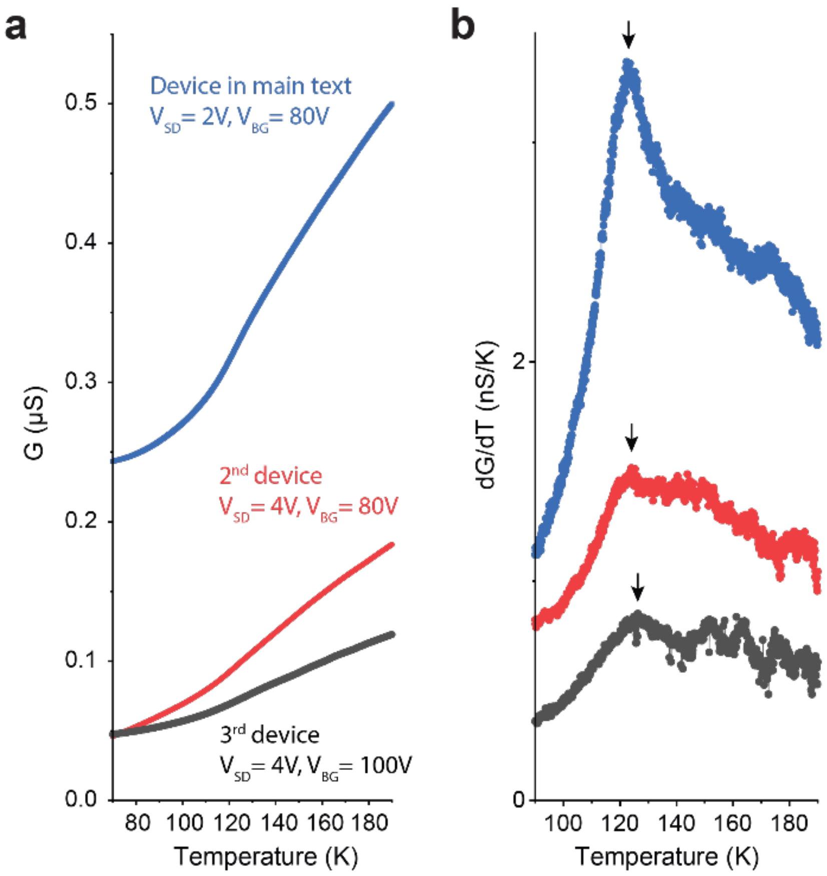

Figure S2. **Conductance measurements of additional 1L $NiPS_3$. a–b.** Conductance (**a**) and its temperature derivative (**b**) as a function of temperature for three monolayer (1L) devices: the device shown in the main text (blue), and two additional devices (2nd in red and 3rd in black). Black arrows indicate the temperatures at *dG/dT* peaks, which are at 124 ± 1 K.

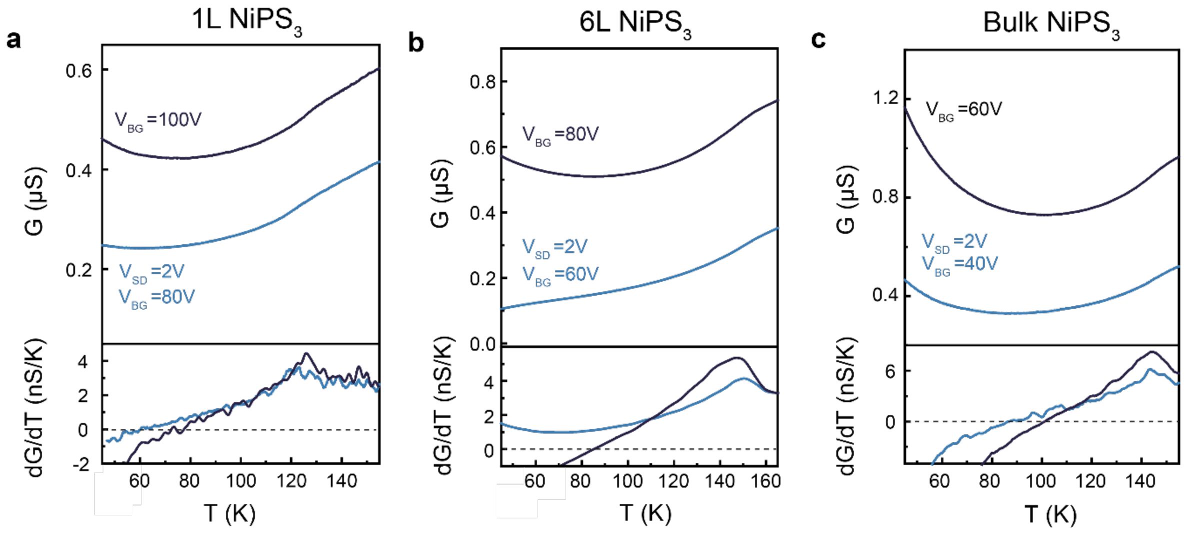

Figure S3. a-c. *G* (top panel) and *dG/dT* (bottom panel) as a function of temperature for 1L (**a**), 6L (**b**) and bulk (> 20L) (**c**) $NiPS_3$, measured at different gate voltages. The position of peak in *dG/dT* does not show gate voltage dependence and corresponds to a magnetic phase transition (**Figure 1c, d**). Contrary, the temperature at which *dG/dT*=0 strongly depends on the applied gate voltage, and therefore should not be related to a magnetic phase transition.

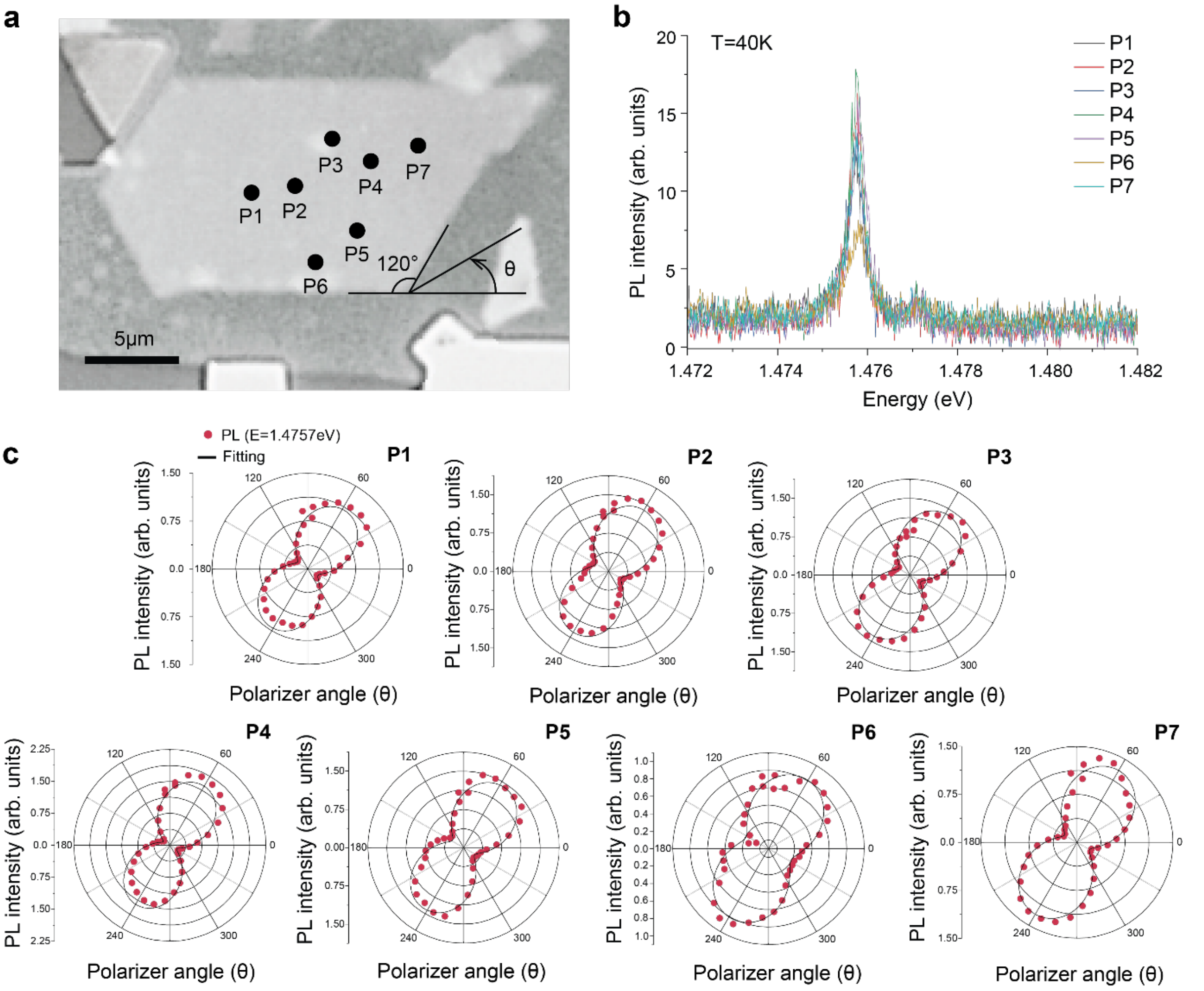

Figure S4. **Spatial orientation of Néel vector in 6L $NiPS_3$ sample probed by polarization-resolved photoluminescence. a.** Black-and-white image of 6L $NiPS_3$ device. Black circles (labeled P1, P2, … P7) correspond to the laser spots (area of ~ 1 μm^2) where PL spectra were taken; θ is the angle of the detector linear polarizer from the edge of $NiPS_3$ crystal. **b.** PL spectra from the spots shown in **a** at T=40 K, all of which show the sharp emission around E=1.4757 eV. All the spectra are taken with fixed θ=65°. **c.** Polar plots of PL as a function of θ from the spots shown in **a**. The black lines correspond to the sinusoidal fittings, on average resulting in polarization angle θ_P = 59 ± 2°. The data were collected during two distinct cooldowns, which highlights the reproducibility of the magnetic order during thermal cycling (see also **Figure S5**).

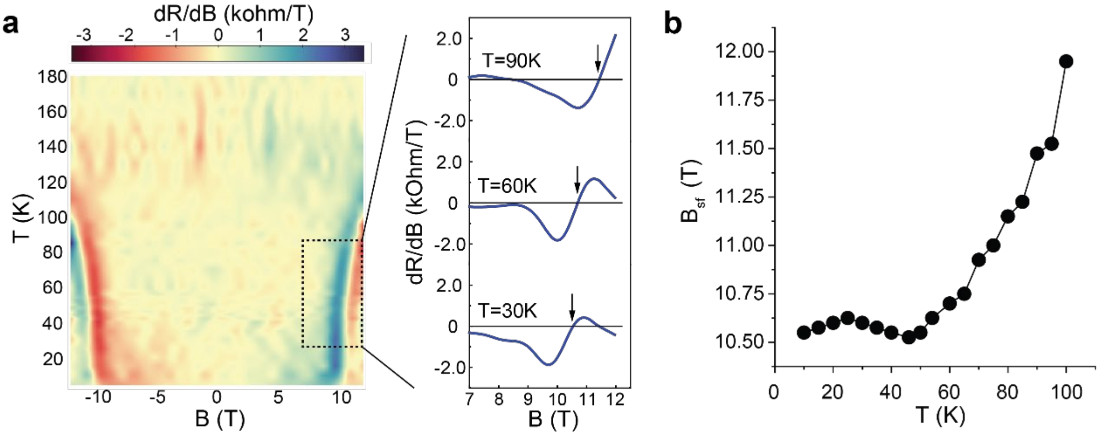

Figure S5. Spin-flop field approximation from resistance derivative (dR/dB). a. The left panel is 2D plot of *dR/dB* as a function of magnetic field and temperature for 6L $NiPS_3$ from the data in figure 2d in the main text. The right panel shows *dR/dB* in the *B*=7-12 T range for *T*= 30 K, 60 K and 90 K. Black arrows indicate the fields corresponding to *dR/dB*=0, which are taken as the estimated spin-flop fields (B_{sf}). **b**. B_{sf} as a function of temperature.

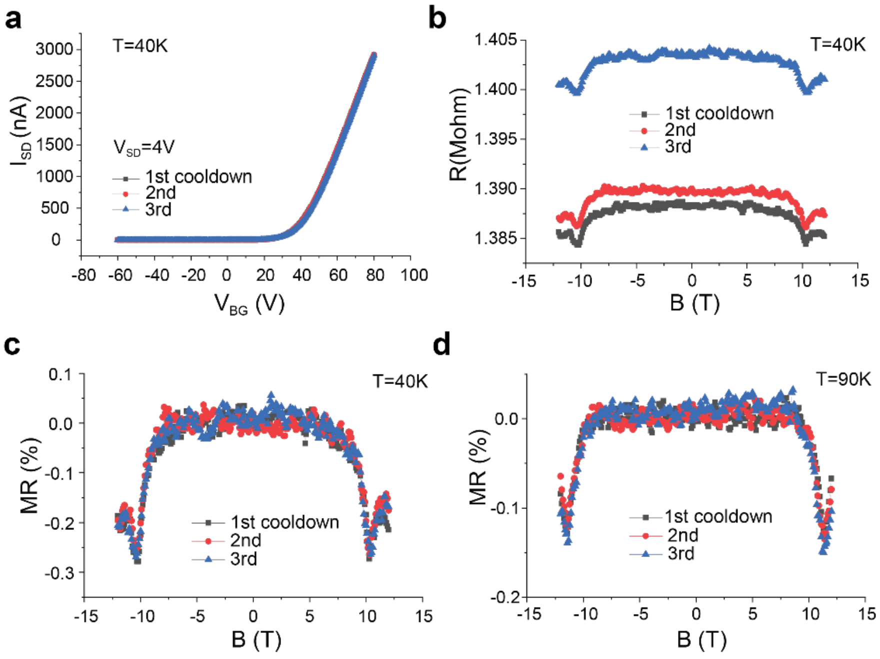

Figure S6. Reproducibility of MR in multiple thermal cycles. a. Transfer curves of 6L $NiPS_3$ measured at *T*=40 K with V_{SD}=4 V and V_{BG}=80 V from three separate cooldowns from *T*=300 K. **b-d.** Resistance and *MR* as a function of the magnetic field with **B** along **L** at *T*=40 K (**b, c**) and 90 K (**d**) from the three separate cooldowns.

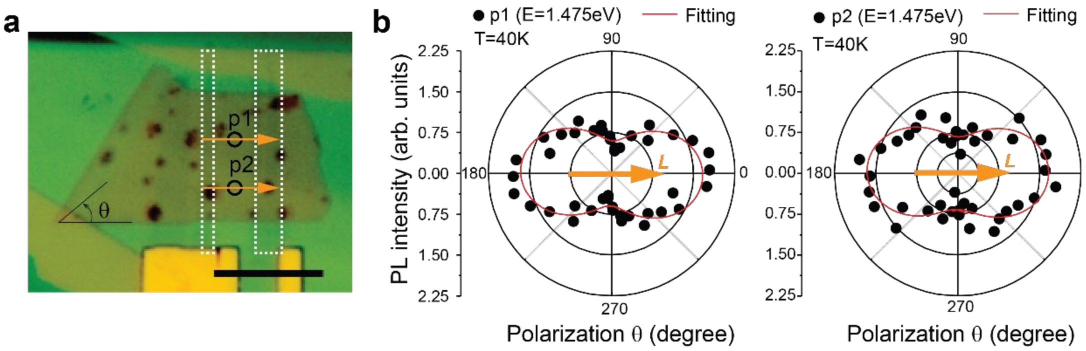

Figure S7. **Spatial orientation of Néel vector in 13L $NiPS_3$ sample probed by polarization-resolved photoluminescence. a**. Optical microscopy image of a 13L $NiPS_3$ FET device. The optical excitation areas are illustrated by open circles (p1 and p2) located between the two few-layer graphite strips (outlined by white dashed line). The scale bar is 5 μm. The angle θ denotes the orientation of the detector linear polarizer with respect to the edge of $NiPS_3$ crystal. **b**. Polar plots of PL intensity (E=1.475eV at T=40K) as a function of θ (left panel from p1; right panel from p2). The Neel vector (**L**, orange arrow) is oriented along the edge of the crystal shown in **a**, consistent with the orientation observed in 6L (see **Fig. S3**).

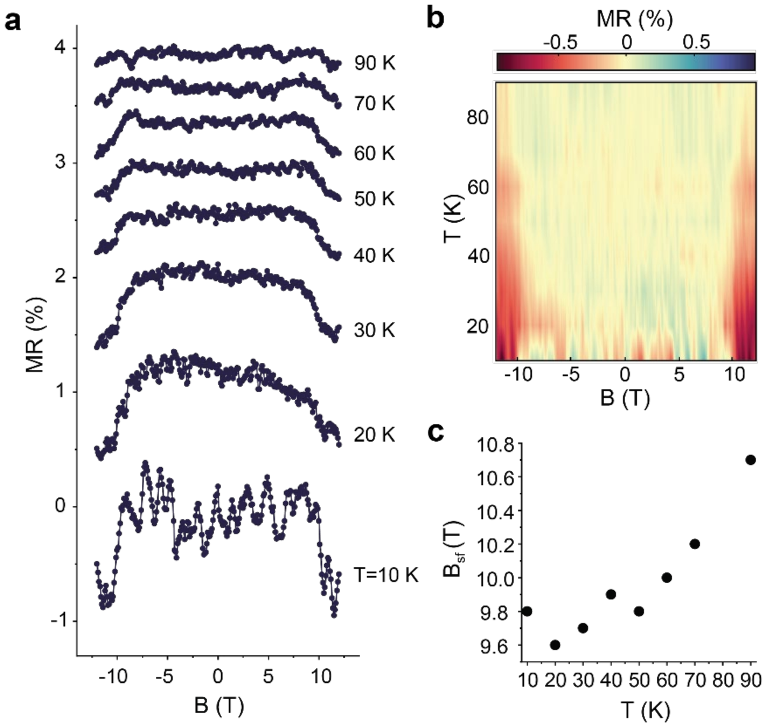

Figure S8. Temperature dependent MR of 2L $NiPS_3$. a. MR of 2L $NiPS_3$ at various temperatures measured as a function of in-plane magnetic field along the easy axis. Measurements are done with fixed V_{SD}=4V and V_{BG}=40V. Data are presented with vertical offsets for clarity. **b.** 2D plot of MR from (**a**). **c**. Extracted B_{sf} as a function of temperature. At each temperature, B_{sf} is estimated from the magnetic field corresponding to the minimum of dR/dB.

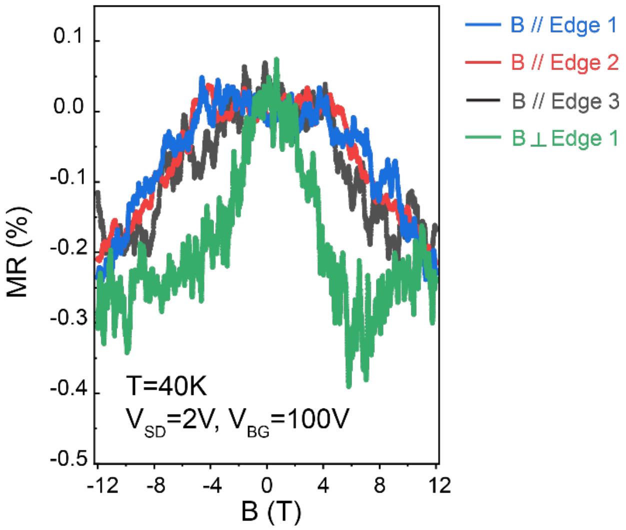

Figure S9. MR data for 1L $NiPS_3$ device measured with an in-plane field perpendicular to edge #1 (green), compared to the data from **Figure 3c**.

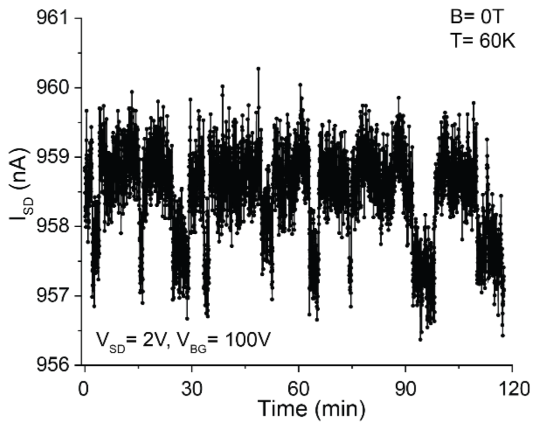

Figure S10. Time trace of electrical current 1L $NiPS_3$ at 60 K without magnetic field. Fluctuations of the electrical current indicate the switching, which is independent of applied magnetic field.

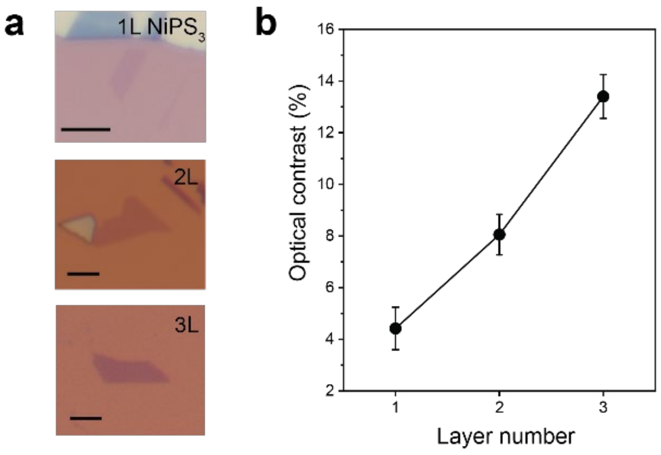

Figure S11. **Thickness characterization of few-layer $NiPS_3$ based on optical contrast**. **a-b**. Optical microscopy image (**a**) and the optical contrast (%) from the red channel (**b**) of 1L, 2L and 3L $NiPS_3$. The scale bar in **a** is 5 μm. The error bar in **b** represents the standard deviation of the contrast values collected from multiple samples.
